# Occupational stress and its related demographic factors among Iranian CCU nurses: a cross-sectional study

**DOI:** 10.1186/s13104-019-4674-5

**Published:** 2019-09-27

**Authors:** Azam Faraji, Mahtab Karimi, Seyyed Mohsen Azizi, Maryam Janatolmakan, Alireza Khatony

**Affiliations:** 1 Clinical Research Development Center of Imam Reza Hospital, Kermanshah University of Medical Sciences, Kermanshah, Iran; 2 Student Research Committee, Kermanshah University of Medical Sciences, Kermanshah, Iran; 3 Clinical Research Development Center of Imam Reza Hospital, Kermanshah University of Medical Sciences, Kermanshah, Iran; 4 Health Institute, Social Development and Health Promotion Research Center, Kermanshah University of Medical Sciences, Kermanshah, Iran

**Keywords:** Critical care unit, Demographic factors, Nurse, Occupational stress

## Abstract

**Objectives:**

Occupational stress can have an adverse effect on mental and physical health and performance of nurses. The aim of this study was to investigate the occupational stress of Iranian critical care unit (CCU) nurses and its related demographic factors.

**Results:**

In this cross-sectional study, 155 CCU nurses were randomly selected. The Osipow Occupational Stress Questionnaire was used as data collection tool. The mean of nurses’ occupational stress was 210.13 ± 40.87 out of 300, which was at the “moderate-to-high” level. The highest mean of occupational stress was related to the subscale of “Role Overload” (36.30 ± 6.98) and the lowest mean was related to the subscale of “Physical Environment” (33.58 ± 9.76). There was no statistically significant difference between the mean occupational stress and variables of sex, age, academic degree and working experience.

## Introduction

Nursing is recognized as one of the most stressful professions [1, 2]. Occupational stress can have an adverse effect on mental and physical health and performance of nurses [3]. It can also cause low self-confidence, low productivity and job dissatisfaction [4, 5]. Occupational stress refers to a condition in which, the factors associated with the job would change the psychological and physiological conditions of an individual and cause the person to diverge from normal functioning [6]. In a general division, occupational stress consists of six dimensions including; role ambiguity, responsibility, physical environment, role insufficiency, role boundary, and role overload [7]. In the nursing profession, due to the specific nature of this profession, there are various stress factors that can have an adverse effect on individuals and the organizations. Stress factors are related to work organization, financial resources and communication [8]. Based on the results of past studies, psychological and physical abuse and violence, exposure to death, shortage of personnel, high number of patients, being exposed to infection, and conflict with nursing managers are common stressors in the work environment of nurses [5, 9]. Identification and management of factors that can cause occupational stress in nurses is essential. One of the important measures that should be taken by hospitals to improve the working conditions and reduce the work stress of nurses is to examine the occupational stress and its related factors [10–12]. Several studies have been conducted on occupational stress of nurses. In a study, the Greek nurses’ occupational stress was measured to be at moderate level [13]. Result of another study indicated a moderate level of occupational stress among Ugandan nurses [14]. Based on the results of a study, nurses from remote areas of Australia had a high level of stress and psychological distress [15]. The results of a study in Ireland also revealed a high level of occupational stress among Irish nurses [16].Considering the prevalence of occupational stress among nurses and the negative consequences of occupational stress [1, 9, 17], and also due to the lack of knowledge about occupational stress and its related factors among nurses affiliated to the Kermanshah University of Medical Sciences (KUMS), the present study was carried out to investigate the occupational stress and its related demographic factors in critical care unit (CCU) nurses.

## Main text

### Methods

#### Study questions

We sought to answer the following questions: (1) What is the level of occupational stress of Iranian nurses?, (2) Is there a significant difference between the mean of occupational stress and demographic factors of nurses?.

#### Study design

This cross-sectional study was conducted on Iranian nurses during July to August 2018. The CCU nurses working in hospitals affiliated to KUMS were the statistical population. Using the Cochran’s sample size formula, the sample size was set to be 155 individuals. The samples were selected using a stratified random sampling method. The stratum of sampling was the hospitals affiliated to KUMS. In each stratum, a simple randomized sampling was done using a random table of numbers. The inclusion criteria were willing to participate in the study, having at least a bachelor’s degree in nursing, and having at least 1 year of work experience in the CCU.

#### Instrument

Data collection was done using the individual information form and Osipow Occupational Stress Questionnaire (OOSQ). The individual information form included questions about sex, age, academic degree, and work experience of nurses. Validity and reliability of the Persian version of OOSQ have been confirmed in Iranian studies [4, 7]. The reliability of this questionnaire has been reported to be 0.88 using the Cronbach’s Alpha coefficient [18]. The OOSQ usually includes 6 items and 6 sub-scales including role boundary, role overload, responsibility, role insufficiency, role ambiguity, and physical environment. Each item is scored based on the 5-point Likert scale (1 = never, 2 = occasionally, 3 = sometimes, 4 = usually, and 5 = most of the time). The higher score in this scale indicates the higher level of occupational stress. Scores of the questionnaire’s subscales are categorized in 4 levels, including low (10–19), low-moderate (20–29), moderate-high (30–39), and high (40–50). The total score of the questionnaire is also categorized into 4 levels, including, low (60–119), low-moderate (120–179), moderate-high (180–239), and high (240–300) [7].

#### Data collection

For data collection, the researcher attended the hospitals affiliated to the KUMS and obtained a list of nurses working in the CCU of each hospital. The list was numbered and then the samples were selected based on the random number table. In the next step, the researcher contacted the nurses according to their work schedule. First, the goals of the study were explained to the nurses and if they were satisfied, they were asked to participate in the study.

#### Data analysis

The data were analyzed by the Statistical Package for Social Sciences (SPSS v.16.0; SPSS Inc., Chicago, IL, USA) using descriptive and analytical statistics. First, the Kolmogorov–Smirnov test was performed, which resulted in the normal distribution of occupational stress. In the descriptive statistics, frequency, percentage, mean and standard deviation were used. Independent sample t-test, one-way ANOVA and Pearson correlation coefficient were also used in inferential statistics. The Independent sample t-test was used to compare the mean of occupational stress in terms of dual-mode qualitative variables (sex, academic degree), and the ANOVA test was used to compare the mean of occupational stress in terms of multi-mode qualitative variables (work experience, age groups). Pearson correlation test was used to examine the relationship between the sub-scales of occupational stress and total score of occupational stress. The significance level of 0.05 was considered.

#### Ethical considerations

The study was approved by the University’s Ethics Committee with the code: IR.KUMS.REC.1396.413. The aims of the study were explained to the participants and a written informed consent was obtained from all of them.

### Results

From the 155 statistical samples, the majority were women (n = 119, 76.8%) and had B.Sc. degree (n = 124, 80.0%). The mean age and work experience of nurses were 34.52 ± 5.07 and 11.27 ± 12.57 years, respectively (Table 1).

**Table 1:** Comparison of occupational stress in term of emographic characteristics

Variables	n (%)	Mean ± SD^a^	Test results
Sex			
Female	119 (76.8)	209.20 ± 40.29	t = − 0.516 *p *= 0.529
Male	36 (23.2)	213.22 ± 43.14	
Academic degree			
B.Sc.	124 (80.0)	208.14 ± 39.09	t = − 1.214 *p *= 0.062
M.Sc.	31 (20.0)	218.09 ± 47.16	
Age (years)			
20–30	34 (21.9)	197.00 ± 39.19	F = 2.294 *p *= 0.104
31–40	107 (69.0)	213.98 ± 39.58	
≥ 41	14 (9.0)	212.64 ± 50.28	
Age (years), mean (SD)	34.52 (5.07)		
Working experience (years)			
1–5	37 (23.9)	202.81 ± 47.36	F = 0.735 *p *= 0.533
6–10	42 (27.1)	208.76 ± 36.07	
11–15	55 (35.5)	213.41 ± 40.74	
≥ 16	21 (13.5)	217.19 ± 38.58	
Working experience (years), mean (SD)	11.27 (12.57)		

^a^Standard deviation

The total mean score of nurses’ occupational stress was 210.13 ± 40.87 out of 300, which was at the moderate-to-high level. Occupational stress of about half of the nurses (n = 77, 49.7%) was at moderate-to-high level. Among the subscales of occupational stress, the highest and lowest mean were related to the subscales of role overload (36.30 ± 6.98) and physical environment (33.58 ± 9.76), which were at medium-to-high level (Table 2).

**Table 2:** Frequency and mean of occupational stress and its sub-scales among nurses

	Role overload	Role insufficiency	Role ambiguity	Role boundary	Responsibility	Physical environment	Total occupational stress score
	n (%)	n (%)	n (%)	n (%)	n (%)	n (%)	n (%)
Low	–	2 (1.3%)	3 (1.9%)	3 (1.9%)	8 (5.2%)	12 (7.7%)	1 (0.6%)
Low-moderate	25 (16.1%)	46 (29.7%)	28 (18.1%)	39 (25.2%)	38 (24.5%)	42 (27.1)	38 (24.5%)
Moderate-high	71 (45.8%)	60 (38.7%)	67 (43.2%)	61 (39.4%)	54 (34.8%)	51 (32.9%)	77 (49.7%)
High	59 (38.1%)	47 (30.3%)	57 (36.8%)	52 (33.5%)	55 (35.5%)	50 (32.3%)	39 (25.2%)
Mean ± SD^a^	36.30 ± 6.98	34.55 ± 7.84	36.03 ± 7.40	34.90 ± 7.97	34.75 ± 8.42	33.58 ± 9.76	210.13 ± 40.87
95% CI^b^	34.14–37.34	33.27–35.75	34.83–37.25	33.60–36.13	33.41–36.05	31.90–35.10	203.72–216.85

^a^Standard deviation

^b^Confidence interval

The mean occupational stress in male nurses (213.22 ± 43.14) was higher than female nurses (209.20 ± 40.29). However, this difference was not statistically significant. The nurses with the M.Sc. degree had a higher mean of occupational stress than the nurses with B.Sc. degree (218.09 ± 47.16 and 208.14 ± 39.09 respectively), but this difference was not statistically significant (Table 1). The nurses that were in the age group of 31–40 years had a higher mean of occupational stress (213.98 ± 39.58) compared to other age groups, but this difference was not statistically significant.

The mean of occupational stress in nurses with the work experience of more than 16 years was higher than other nurses (217.19 ± 38.58), however this difference was not statistically significant (Table 1).

There was a positive and significant relationship between the total score of occupational stress and its subscales at the level of P ≤ 0.01. The highest and lowest correlations were found between the mean occupational stress and subscales of role boundary (r = 0.904, P ≤ 0.01) and physical environment (r = 0.766, P ≤ 0.01), respectively (Table 3).

**Table 3:** Correlation between occupational stress and its sub-scales

Variable		1	2	3	4	5	6	7
1	Role overload	1.000						
2	Role insufficiency	0.614**	1.000					
3	Role ambiguity	0.706**	0.722**	1.000				
4	Role boundary	0.748**	0.708**	0.776**	1.000			
5	Responsibility	0.648**	0.702**	0.756**	0.767**	1.000		
6	Physical environment	0.561**	0.471**	0.552**	0.614**	0.589**	1.000	
7	Total occupational stress score	0.830**	0.823**	0.880**	0.904**	0.879**	0.766**	1.000

**P ≤ 0.01

### Discussion

In our study, the mean occupational stress in nurses working in CCU was moderate to high. The highest and lowest mean of occupational stress, were related to the subscales of role overload and physical environment, respectively. In regard to the level of occupational stress in nurses, the results of some studies are consistent with the findings of our study [13–16]. In this regard, the results of a study showed that Chinese nurses had high level of occupational stress [19]. Also, in a study done on Botswana nurses, a high percentage of nurses had occupational stress [20]. The high mean of occupational stress in nurses in this study can be due to the sensitive condition of CCUs. In the CCU, nurses experience a high level of stress because of the critical condition of patients, and CCU nurses are required to have flexibility and capability to respond quickly in critical situations as compared with other nurses. Also, alertness is another feature that nurses in the CCU should have. In addition to the characteristics and conditions governing the CCU, other factors can also contribute to the occupational stress of CCU nurses. In this regard, evidence suggests that factors such as workload, physical environment, and ambiguity of duties can also contribute to the high level of occupational stress among CCU nurses [21]. In this study, the highest mean of occupational stress was related to the role overload. Therefore, reducing workload and improving nurses’ environmental condition can play a significant role in reducing their job stress [22].

We did not find any statistically significant difference between the nurses’ mean occupational stress and their sex. In some studies, there was no relationship between the mean of occupational stress of nurses and their sex [23, 24]. However in a study done on Iranian nurses, the difference in the mean of occupational stress in both sexes was significant [25]. In our opinion, occupational stress is a transgender phenomenon and can affect any person, whether male or female.

In our study, nurses with M.Sc. degree had higher level of occupational stress compared to nurses with B.Sc. degree, but this difference was not statistically significant. Our results are in line with the studies of Yim et al. [24] and Sabzi et al. [25], but they are not consistent with the findings of Wu et al. [26] study. In Iran, nurses with a higher level of education are expected to have better performance. On the other hand, nurses with high level of education expect a better performance from themselves than nurses with lower education levels. The expectation of others from nurse and their own expectations from themselves can be effective in creating and increasing job stress in nurses.

In our study, nurses in the age group of 31–40 years had a higher mean of occupational stress compared to other age groups, but this difference was not statistically significant. Our results are in line with the findings of Yim et al., Romano et al., and Sabzi et al. studies [24, 25, 27], but are not consistent with the findings of Wu et al. [26] study. We believe that, the nature of CCU and the health status of patients admitted to CCU can affect all CCU nurses in any age group.

In our study, we did not find any significant difference between the mean of nurses’ occupational stress and their work experience. Sharma et al. and Sahraian et al. also concluded the same finding in their studies [28, 29]. However, Yoosefian Miandoab et al. [30] reported a significant statistical relationship between the mean of occupational stress and work experience, so that with increasing work experience, the nurses’ job stress also increased. Although in our study there was no significant difference between the mean of occupational stress and work experience of nurses, we believe that with the increase in work experience, nurses’ occupational stress is expected to decrease. In our opinion, one of the most important factors that can lead to a reduction in the level of occupational stress of nurses with high work experience is the clinical competence that over time increases through the acquisition of various occupational experiences in the field of patient care.

## Limitations

One of the limitations that could affect the outcome of this study was psychological condition of the samples when completing the questionnaires. Although the researcher tried to refer to the samples at the right time to complete the questionnaire, the full control of this issue was beyond our control. The second limitation was the cross-sectional method of this study, which does not examine the causal relationship between the study variables.

## Data Availability

The datasets used during the current study are available from the corresponding author on reasonable request.

## Abbreviations

KUMS: Kermanshah University of Medical Sciences
OOSQ: Osipow Occupational Stress Questionnaire
CCU: Critical Care Unit
SPSS: Statistical Package for Social Sciences
